# Niobium Modification of Au/CeO_2_ for Enhanced Catalytic Performance over Benzene Combustion

**DOI:** 10.3390/nano11010189

**Published:** 2021-01-13

**Authors:** Zhe Liu, Xiaolan Zhang, Ting Cai, Jing Yuan, Kunfeng Zhao, Wenquan Lu, Dannong He

**Affiliations:** 1 School of Materials Science and Engineering, Shanghai Jiao Tong University, Shanghai 200240, China; hexor000@sjtu.edu.cn (Z.L.); zhangxiaolan@sjtu.edu.cn (X.Z.); Jiangmuyang@sjtu.edu.cn (T.C.); zhaokunfeng-gl@163.com (K.Z.); 2 National Engineering Research Center for Nanotechnology, Shanghai 200241, China; hdn_nercn@163.com

**Keywords:** niobium modification, Au/Nb-CeO_2_, benzene, catalytic combustion

## Abstract

A novel Au/Nb-CeO_2_ was obtained by loading Au to Nb-modified CeO_2_ adopting a thermal decomposition method. The modification effect of Nb on the physicochemical properties and performance of Au/CeO_2_ for benzene combustion was systematically clarified. The incorporated Nb species are found to be present in the two forms of highly-dispersed state and bulk NbO*_x_* into CeO_2_ lattice in the obtained Au/Nb-CeO_2_ catalyst. They greatly enlarged the BET surface area, improved the redox property, and strengthened the Au–support interaction. The addition of Nb also promotes catalytic performance of Au/CeO_2_, especially high-temperature performance: *T*_90%_ decreases by ca. 40 °C and Au/Nb-CeO_2_ exhibits superior stability to Au/CeO_2_ at 230 °C. The slightly improved Au dispersion and redox properties resulted in the small increase on initial activity of Au/Nb-CeO_2_, but the large BET surface area and the strong Au–support interaction greatly promoted the high-temperature performance improvement of Au/Nb-CeO_2_ for benzene combustion reaction.

## 1. Introduction

Environmental pollution caused by volatile organic compounds (VOCs) have great harm to human health [1,2]. Therefore, VOCs pollution control are imperative. Up to now, catalytic combustion for VOCs control has been the important research subject in environmental management field, among which heterogeneous catalysis study on the building of high-performance catalysts for benzene combustion reaction is of great significance for alleviating air pollution [3,4,5].

Au/CeO_2_-based catalytic materials have attracted extensive attention owing to excellent low-temperature catalytic ability of nano-Au [6,7,8,9] and strong oxygen storage capacity of cerium oxide [10,11,12]. There are also many reports devoting to improving its catalytic performance, mainly by modifying the properties of CeO_2_ [13,14,15,16]. Among them, metals doping to CeO_2_ [17,18] is reported to be an effective strategy, especially in catalytic oxidation reactions, since proper metal dopants can promote oxygen mobility of CeO_2_ and stabilize nano-Au. Au/V doped-CeO_2_ usually exhibited excellent catalytic performance among the derived Au/CeO_2_ catalysts [15,16]. However, the toxicity of V further limits their application. Therefore, it is urgently needed to explore potential human-friendly candidate dopants to enhance the catalytic performance of Au/CeO_2_ catalyst for benzene combustion.

Fortunately, of the same VB family with V, Nb and its compounds performed beneficially to various catalytic reactions in the past decades, such as hydrocarbon oxidative dehydrogenation, ammonia oxidation, and nitric oxide removal [17,19]. However, only few studies focused on the influences of niobium on Au/CeO_2_ in VOCs removal, as it is hard to guarantee uniformity of components due to difficult solubility of niobium-containing compounds in conventional solvents. Thus, Nb has great potential as an alternative regulator to improve the performance of Au/CeO_2_ catalyst for benzene oxidation once the abovementioned difficulties were overcome.

Herein, the purpose of this work concentrates on the preparation and characterization of a novel Au/Nb-CeO_2_ catalyst. In this work, Nb-CeO_2_ was prepared adopting a thermal decomposition method and then Au was loaded on the Nb-modified CeO_2_ support. The catalytic performances of Au/Nb-CeO_2_ for benzene combustion were then evaluated. Furthermore, the modification effect of Nb on Au/CeO_2_ was systematically studied. This present work will help to explore the application of niobium-containing compounds in VOCs’ removal, and broaden new ideas for optimal design of catalysts.

## 2. Materials and Methods

### 2.1. Sample Preparation

CeO_2_, NbO*_x_*, and Nb-CeO_2_: In brief, an appropriate amount of aqueous solution of Ce(NO_3_)_3_·6H_2_O (0.03 mol/L) and NaOH (0.15 mol/L) were mixed under vigorous stirring at 60 °C for 2 h. Then the precipitates were collected and washed until pH = 7. It was then dried at 70 °C for 12 h and calcined at 400 °C for 2 h to obtain CeO_2_. A certain amount of niobium oxalate were calcinated at 400 °C for 2 h to obtain NbO*_x_*. Nb-CeO_2_ was prepared by a thermal decomposition method. In brief, a certain amount of niobium oxalate and CeO_2_ with molar ratio of Ce:Nb = 15:1 were mechanically mixed for 30 min, then calcined at 400 °C for 2 h to obtain Nb-CeO_2_.

Au/CeO_2_, Au/Nb-CeO_2_, and Au/NbO*_x_*: 1 wt% Au/CeO_2_ and Au/Nb-CeO_2_ was synthesized by a deposition-precipitation method. In brief, 1 g of CeO_2_ or Nb-CeO_2_ powder was added to an appropriate amount of aqueous solution of HAuCl_4_ (containing 0.01 g Au) under vigorous stirring at 60 °C. 0.05 mol/L NaOH solution was added into the suspension solution to adjust pH to 9. Then the obtained solution was kept stirring for 2 h at 80 °C. The precursor was collected, washed, dried under vacuum at 70 °C for 12 h and then calcined at 400 °C for 2 h to obtain Au/CeO_2_ or Au/Nb-CeO_2_ catalyst. Au/NbO*_x_* (Au loading was 1 wt %) was prepared by an impregnation method. In brief, 1 g of NbO*_x_* powder obtained by directly calcined niobium oxalate was added to aqueous solution of HAuCl_4_ (containing 0.01 g Au) under vigorous stirring at 80 °C. Then the suspension was kept stirring until water was completely evaporated. The obtained precursor was thoroughly washed with dilute ammonia solution, and then dried under vacuum at 70 °C for 12 h and calcined at 400 °C for 2 h to obtain Au/NbO*_x_* catalyst.

### 2.2. Catalyst Characterization

The X-ray diffraction (XRD) patterns were recorded on a D/MAX-2600PC diffractometer (Rigaku, Science of Japan Co., Ltd., Tokyo, Japan) operated at 40 kV and 100 mA with a scanning angle (2θ) of 20–90° at a scanning speed of 5 °/min. The BET specific surface of the prepared samples were determined by adsorption–desorption of N_2_ at liquid nitrogen temperature using a Micromeritics ASAP 2010 analyzer (Micromeritics, Norcross, GA, USA). The actual mass fraction of the Au in as-prepared catalysts was determined by inductive coupled plasma (ICP) on an iCAP7600 instrument (Thermo Fisher Scientific, Waltham, MA, USA). Transmission electron microscopy (TEM), high resolution transmission electron microscope (HRTEM), and scanning TEM images were performed on a JEM-2100 (JEOL) analyzer at an accelerating voltage of 200 kV. Raman spectra were recorded using an inVia reflex laser Raman instrument equipped with a charge coupled device (CCD) detector (Renishaw, Pliezhausen, Germany). The excitation source was the 514.5 nm line of Ar ions laser at a laser power of ca. 3 mW. H_2_-TPR experiments and CO pulse adsorption were carried out on an Auto Chem II chemisorption 2920 analyzer (Micromeritics, Norcross, GA, USA) apparatus equipped with a thermal conductivity detector (TCD). H_2_ temperature programming reduction (H_2_-TPR) experiments were carried out by heating catalysts (100 mg) in 10% H_2_-Ar flow (30 mL/min) at a heating rate of 10 °C/min from 40 to 900 °C. Before CO pulse adsorption experiment, 100 mg of catalyst was reduced at 300 °C for 2h in 10% H_2_-Ar atmosphere and then cooled to 50 °C, Next, the catalyst was swept with a He flow (50 mL/min). Finally, the catalyst was purged with a 10% CO-He flow. XPS measurements were made on an Axis Ultra DLD (Kratos, Hadano, Japan) spectrometer with a monochromatic Al K_α_ radiation (1486.6 eV). The charging effect of samples was corrected by setting the binding energy of adventitious carbon (C 1s) at 284.6 eV.

### 2.3. Catalytic Activity Measurements

The catalytic activity measurements for the catalytic oxidation of benzene were carried out at an ambient pressure in a quartz tubular fixed bed reactor (ID 8 mm), where 80 mg of catalyst (40–60 mesh) was placed. A thermocouple to monitor the reaction temperature was fixed near the reactor. The catalytic reaction was carried out from 80 °C to 400 °C. The inlet flow to the reactor was 40 mL/min with the weight hourly space velocity (WHSV) of 30,000 mL g^−1^ h^−1^. The feed gas was composed of benzene 1000 ppm balanced with air. The gaseous reactant of benzene was generated by causing N_2_ to flow into a vapor saturator. For comparison, the related activity tests were also carried out under different conditions. The concentration of effluent gas was analyzed by a gas chromatograph (Varian GC-450) (Varian Inc., Palo Alto, CA, USA) equipped with a flame ionization detector (FID). The yield to CO_2_ was measured using a mass spectrometer, HPR20 model (Hiden, Warrington, UK).

## 3. Results and Discussion

**Figure 1:**
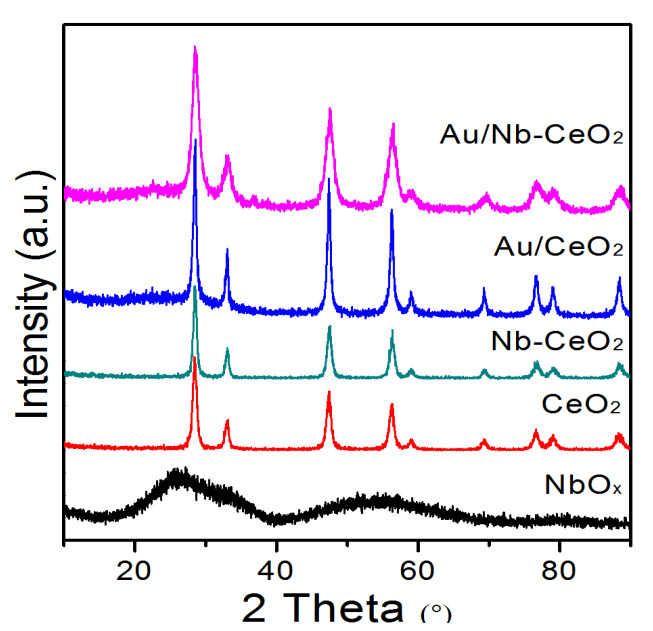
XRD patterns of Au/Nb-CeO_2_, Au/CeO_2_, Nb-CeO_2_, CeO_2_, and NbO*_x_*.

Figure 1 shows XRD patterns of as-prepared catalysts. NbO*_x_* presents amorphous structure whereas CeO_2_ exhibits a typical cubic fluorite structure (JCPDS no. 81-0792) [20]. After Nb was added to CeO_2_, Nb-CeO_2_ presented the almost same structure as CeO_2_. When Au was loaded on CeO_2_ and Nb-CeO_2_, both Au/CeO_2_ and Au/Nb-CeO_2_ also mainly exhibit CeO_2_ crystal structure, whereas no significant diffraction peaks from Au (ca. 38°) were obviously observed in their XRD patterns, indicating the small particle size of gold species on CeO_2_ and Nb-CeO_2_. Additionally, XRD patterns of Nb-CeO_2_ and Au/Nb-CeO_2_ did not show obvious diffraction peaks from NbO_x_, suggesting that Nb-contained species are amorphous or highly dispersed states in these samples. By analyzing the XRD patterns, the lattice parameter of CeO_2_ is slightly larger in Au/Nb-CeO_2_ (5.4163 Å) than in Au/CeO_2_ (5.4127 Å). As Nb*^n^*^+^ is smaller than Ce*^n^*^+^, its partial incorporation into the CeO_2_ lattice is expected, resulting to the expansion of the lattice [21]. Moreover, the decrement of crystalline size of CeO_2_ for Au/Nb-CeO_2_ compared with Au/CeO_2_ (Table 1) indicates that Nb-addition hinders grain growth of CeO_2_. The decreased crystalline size is further confirmed by BET results that Au/Nb-CeO_2_ has a much larger BET specific surface area (203 m^2^ g^−1^) than Au/CeO_2_ (71 m^2^ g^−1^) (Table 1). Also, it is seen from Table 1 that the Au/Nb-CeO_2_ has larger real Au loading than Au/CeO_2_, indicating that Nb dopant is beneficial to the high-dispersion of supported Au particles.

**Figure 2:**
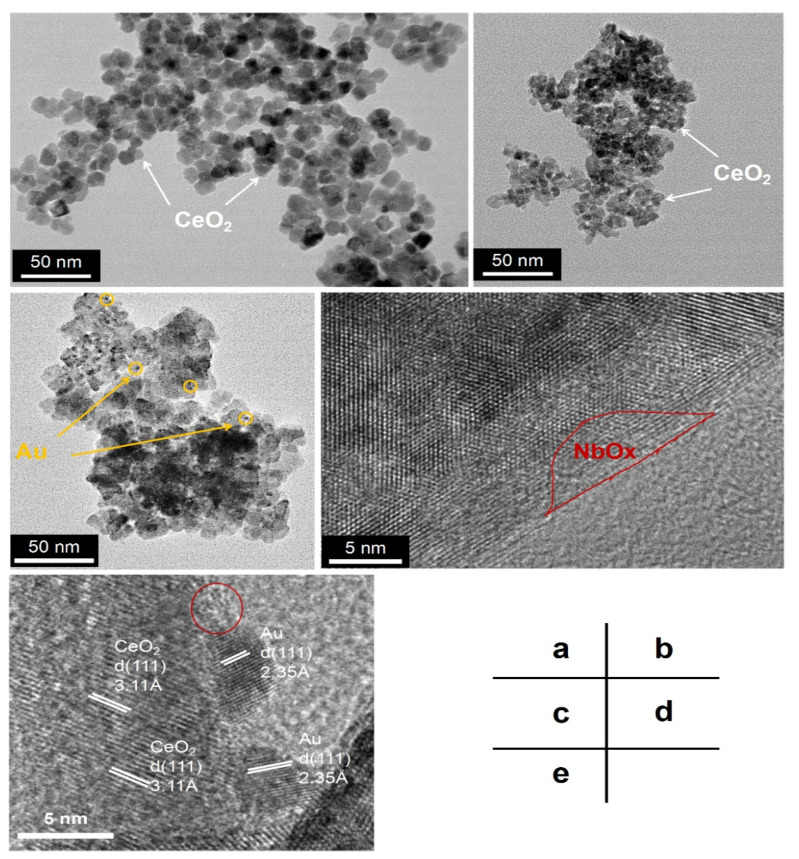
TEM (**a**,**b**), STEM (**c**), and HRTEM (**d**,**e**) images of Au/CeO_2_ (**a**) and Au/Nb-CeO_2_ (**b**–**e**).

Figure 2 shows the microstructure morphology of Au/Nb-CeO_2_ and Au/CeO_2_. Obviously, Au/Nb-CeO_2_ presents smaller CeO_2_ particle size than Au/CeO_2_ (Figure 2a,b), which further confirms XRD results that Nb addition leads in decreased crystalline size. It is also seen from Figure 2c that Au is highly dispersed on Nb-CeO_2_ surface. Additionally, Nb-CeO_2_ support mainly exposes (111) crystal plane of CeO_2_, and there presents amorphous structure (red-marked) affiliated to NbO*_x_* (Figure 2d,e), which are consistent with XRD results. It is worth noting that the amorphous NbO*_x_* seems to have an effect on Au anchoring or interaction between Au and supports (denoted as IMS) from Figure 2e, which will be further elaborated.

**Figure 3:**
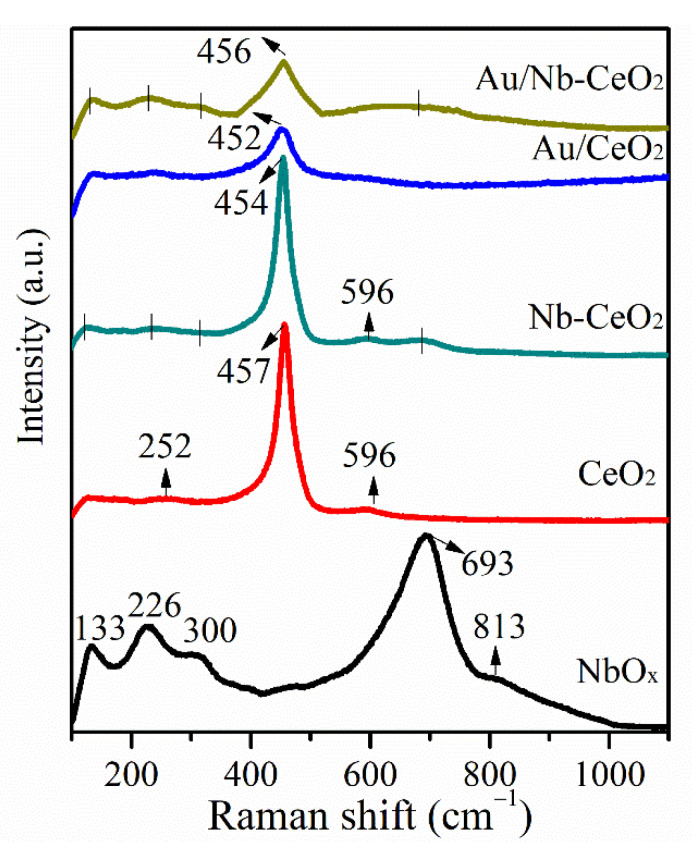
Raman profiles of Au/Nb-CeO_2_, Au/CeO_2_, Nb-CeO_2_, CeO_2_, and NbO*_x_*.

Figure 3 is Raman spectra of as-obtained samples. CeO_2_ exhibits a strong F2g vibration peak at 457 cm^−1^ and a weak oxygen vacancy defect-induced vibrational peak at 596 cm^−1^ [20], while the typical peaks at 133, 226, 300, 693, and 813 cm^−1^ could be associated with NbO*_x_* species [21,22]. Obviously, for Nb-CeO_2_ and Au/Nb-CeO_2_ samples, weak peak at 133, 226, 300, and 693 cm^−1^ are still observed apart from characteristic peak of CeO_2_, indicative of separate NbO*_x_* present in both of them. It is reported that 693 cm^−1^ is assigned to octahedrally coordinated niobium oxide compound [NbO_6_] of Nb_2_O_5_ [22]. In addition, there is a slight red-shift of F2g Raman band from CeO_2_ to Nb-CeO_2_, indicative of a longer Ce-O bond distance with Nb dopant [23]. In the meanwhile, increased band intensity at 596 cm^−1^ is observed on the Raman profile of Nb-CeO_2_, representative of more oxygen vacancies.

Compared with the corresponding ones of Au/CeO_2_, the F2g Raman band of CeO_2_ in Au/Nb-CeO_2_ does not shift to lower frequency as expected in Nb-CeO_2_, but it becomes widened (Table 2). The different modification effect of Nb on the Au/Nb-CeO_2_ and Nb-CeO_2_ might be due to the varied IMS since nano-Au is strongly dependent on support properties [24]. This is in agreement with morphology observation. The widening of F2g band is attributed to increased oxygen vacancies [25,26]. The structural and morphology analysis suggests that some niobium oxide species are present in the form of separate NbO*_x_* species while other niobium oxides are into the lattice of CeO_2_.

**Figure 4:**
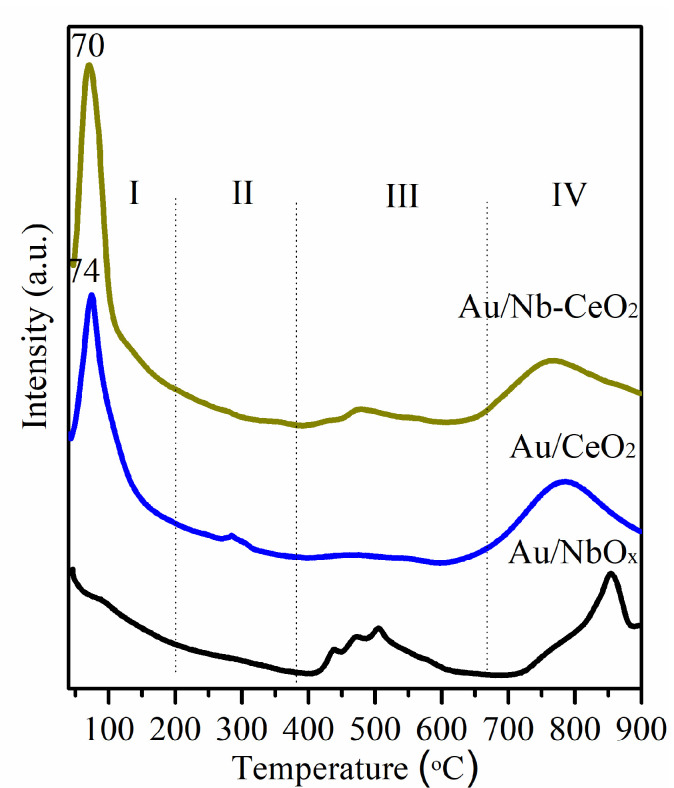
H_2_-TPR profiles of Au/CeO_2_, Au/Nb-CeO_2_, and Au/NbO*_x_*.

Figure 4 presents H_2_-TPR profiles of catalysts, where Au/CeO_2_ and Au/Nb-CeO_2_ exhibit very similar reduction behavior at <200 °C (peak I) and >600 °C (peak IV), whereas a peak in the range 250–350 °C is present in the TPR curve of Au/CeO_2_ (peak II) and another one in the range 400–500 °C in the TPR curve of Au/Nb-CeO_2_ (peak III). It is reported that peak I is ascribed to the reduction of Au*^δ^*^+^ to Au^0^ and to the superficial Ce^4+^ to Ce^3+^, whereas peak II, III, and IV are affiliated to reduction of remaining Ce^4+^ to Ce^3+^ on the surface, superficial Nb^5+^ to Nb^4+^ and reduction of deeper CeO_2_ or NbO*_x_* layers, respectively [27,28]. Compared with Au/CeO_2_, peak II disappears and peak I becomes larger and shifts to lower temperature in the TPR curve of Au/Nb-CeO_2_. Moreover, Au/Nb-CeO_2_ has larger H_2_ consumption amount than Au/CeO_2_ at <200 °C (Table 1). Slightly lowered reduction temperature and increased H_2_ consumption amount are attributed to Nb-promoted surface ceria reduction, showing that incorporated NbO*_x_* species slightly improve the redox ability of surface oxygen species on the modified ceria surface. Additionally, the peak IV shifts to lower temperature over Au/Nb-CeO_2_ than over Au/Nb-CeO_2_, indicating the improvement of the lattice oxygen mobility due to the formation of more oxygen vacancies resulted by NbO*_x_* addition. These are consistent with XRD and Raman results.

**Figure 5:**
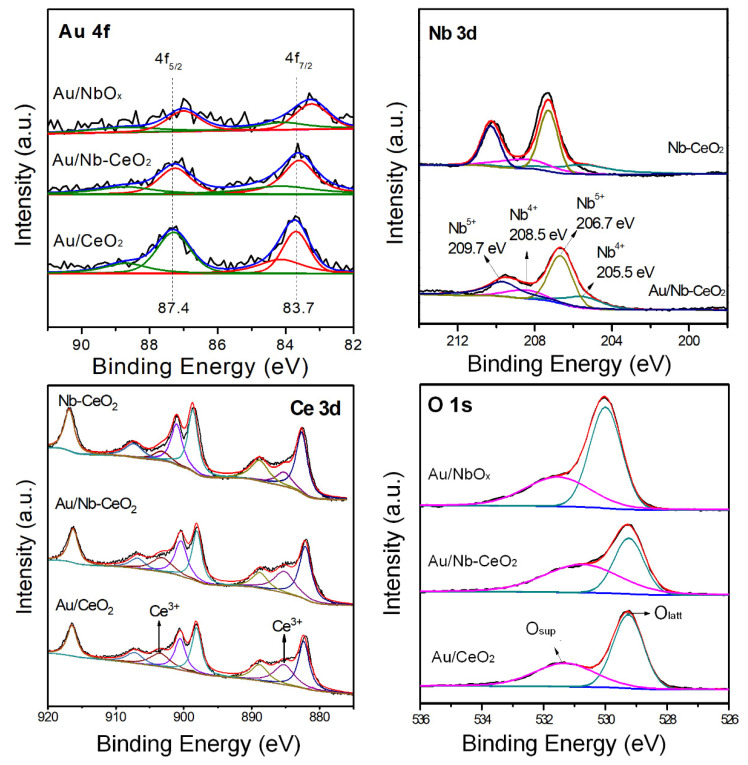
XPS spectra of Au 4f, Nd 3d, Ce 3d, and O 1s for as-obtained samples.

Figure 5 shows the XPS spectra of Au 4f, Nb 3d, Ce 3d, and O 1s for as-obtained samples. It is seen that surficial Nb/Ce molar ratio is 0.37 in Au/Nb-CeO_2_, much larger than 0.07 of ICP result (Table 1), indicating that the majority of NbO*_x_* species are highly dispersed on CeO_2_ surface. Further from the Nb 3d XPS deconvolution of Au/NbO*_x_*, it is revealed that there are two principal signals present at binding energies 206.7 eV and 205.5 eV, characteristic of Nb^5+^ and Nb^4+^, respectively [29,30,31], indicative of two valence of Nb species in Au/Nb-CeO_2_ [20,32]. It is also observed that the obvious deviation of Nb 3d XPS to lower binding energy over Au/Nb-CeO_2_ than Nb-CeO_2_ suggests that Au admission enhances decreased potential energy, and makes valence-electrons migration become much easier.

Both Ce^3+^ (ca. 885.2 eV and 903.2 eV^)^ and Ce^4+^ are present in Nb-CeO_2_, Au/CeO_2_ and Au/Nb-CeO_2_ from Ce 3d XPS curves, and the surface Ce^3+^/Ce^4+^ ratio is slightly higher for Au/Nb-CeO_2_ than for Au/CeO_2_ (Table 1), representative of more oxygen defects on the surface of Au/Nb-CeO_2_. Two fitted peaks with binding energy located at ~530.0 eV and 531.7 eV, correspond to lattice oxygen (O_latt_), and chemisorbed OH species (O_sup_) [33] are observed in the O 1s XPS spectra of Au/Nb-CeO_2_ and Au/CeO_2_, and the former shows higher O_sup_/O_latt_ molar ratio. This suggests that Nb incorporation promoted generation of surface active oxygen species. The Ce 3d and O 1s XPS results are in accordance with TPR and Raman results.

There is a slightly decreased Au 4f_7/2_ BE and increased Au*^δ^*^+^/Au ratio after Nb addition to Au/CeO_2_, indicative of the stronger interaction between Au and Nb-CeO_2_ [34,35]. By comparing the corresponding XPS spectra of Au/Nb-CeO_2_ and Nb-CeO_2_, there is slightly increased surface content of Ce^3+^ and surface active oxygen, contributing to the interaction between Au and Nb-CeO_2_. It is also seen that surface Nb/Ce ratio is 0.67 for Nb-CeO_2_ and 0.37 for Au/Nb-CeO_2_ (Table 1), respectively, indicating that Au admission promoted part of Nb into the CeO_2_ lattice. Thus, Nb addition also generated the strong interaction between Au and Nb-CeO_2_, confirming Raman results and morphology observation.

Thus, according to the data, NbO*_x_* are mainly present in the forms of two states: the majority are highly dispersive on the support surface and the minority are into the lattice. They are largely amorphous and present two valence states, Nb^4+^ and Nb^5+^. Both niobium oxide species modify the properties of Au/Nb-CeO_2_ by lattice distortion and modified interaction between Au and the support. Firstly, as found in XRD, Raman and XPS results, Nb dopant hinders the grain growth of CeO_2_, lengthens Ce-O bond and increases oxygen vacancy. These lead in much larger surface area (203 m^2^ g^−1^ for Au/Nb-CeO_2_ and 71 m^2^ g^−1^ for Au/CeO_2_) and better reducibility of Au/Nb-CeO_2_ than that of Au/CeO_2_ (H_2_-TPR results). Secondly, more small Au particles could be highly dispersed on the surface of modified Nb-CeO_2_ (morphology observation and Au dispersion data). Thirdly, confirmed by Raman and XPS data, compared with Au/CeO_2_, Au/Nb-CeO_2_ exhibited stronger Au–support interaction.

**Figure 6:**
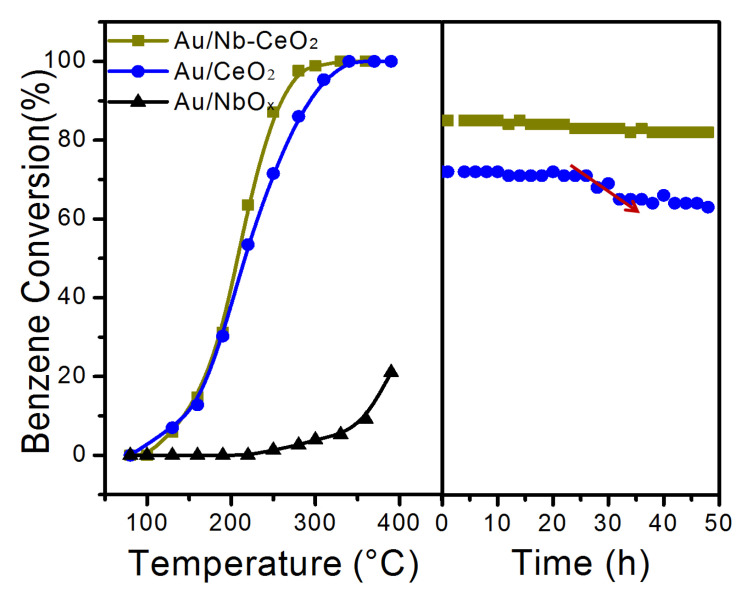
Activity and long-term stability results of as-obtained samples. Reaction conditions for stability test: 230 °C for 48 h, WHSV = 30,000 mL g^−1^ h^−1^, 1000 ppm benzene.

Next, we measured the activity of the novel Au/Nb-CeO_2_ sample for model reaction-benzene total oxidation, and the results are shown in Figure 6. For comparison, the activity test over Au/NbO*_x_* for this reaction was also firstly carried out. CO_2_ and H_2_O were the only products. From Figure 6, under the condition of 1000 ppm benzene and 30,000 mL/(g h) space velocity, Au/NbO*_x_* exhibits a poor activity with *T*_10%_ (the temperature at 10% of benzene conversion) of 358 °C. From Figure 6, there are close *T*_10%_ and *T*_50%_ value for the Au/CeO_2_ and Au/Nb-CeO_2_, but *T*_90%_ of Au/Nb-CeO_2_ decreases by ca. 40 °C than Au/CeO_2_. As the reaction temperature elevated to higher temperature, increasing activity increase was shown for the two samples. The *T*_10%_, *T*_50%_, and *T*_90%_ for Au/CeO_2_ and Au/Nb-CeO_2_ are 147 °C, 215 °C, and 294 °C; and 144 °C, 207 °C, and 258 °C, which decreased by 3 °C, 8 °C, and 36 °C after Nb addition, respectively. That is, large activity improvement turned up at relatively high reaction temperature for benzene oxidation, especially after reaction temperature was elevated to above 200 °C. Thus, the Nb addition improved the catalytic activity of Au/CeO_2_, especially the high-temperature activity. Further the high-temperature performance improvement was verified by the long-term reaction at 230 °C. After 48 h of on-streams reaction at 230 °C, there is hardly any activity loss on Au/Nb-CeO_2_, whereas the conversion rate of Au/CeO_2_ begins to decrease obviously when benzene oxidation lasts for 20 h, indicating that Au/Nb-CeO_2_ presents much better long-term stability than Au/CeO_2_ at 230 °C.

Au dispersion of the fresh and the used Au/CeO_2_ and Au/Nb-CeO_2_ samples were further measured to analyze the reason for the above performance difference, which are shown in Table 3. Obviously, the Au dispersion is slightly higher for the fresh Au/Nb-CeO_2_ (72%) than Au/CeO_2_ (68%). Their *TOF*_Au_ value was further calculated over moles of the surface Au atoms at 160 °C (at which the conversion is below 15%, where mass transfer limitation can be ignored). *TOF*_Au_ values are 54.6 s^−1^ for Au/Nb-CeO_2_ and 53.4 s^−1^ for Au/CeO_2_, showing that nano-Au has approximately same catalytic capacity in the fresh two samples. After 1st run, the Au dispersion for Au/CeO_2_ decreased by 14% and the Au particle size grows by 0.43 nm, while no obvious Au dispersion and particle size growth were observed for the Au/Nb-CeO_2_-1st and even Au/Nb-CeO_2_-48 h (Table 3). That is, Au nanoparticles in Au/CeO_2_ tend to aggregate with each other but they could always be stabilized on the surface of Au/Nb-CeO_2_ during the benzene oxidation process. Even at high-temperature, the small Au nanoparticles could still be highly-dispersed on the Au/ Nb-CeO_2_ surface. These results demonstrated that the Nb addition are beneficial to keep the Au stabilization on the support surface.

It was reported that the property of gold catalysts is mainly determined by the size of gold particles and the nature of support [36,37]. The vacancy/surface lattice oxygen in the support could be the sites of oxygen adsorption/activation through Mars–van Krevelen mechanism. In the present system, compared with Au/CeO_2_, Nb dopant slightly increased the Au dispersion and the redox capacity of CeO_2_, which are the reasons for the small increase on the initial activity over the fresh Au/CeO_2_ and Au/Nb-CeO_2_ for benzene combustion reaction. According to the above characterization results, Nb addition greatly enlarged the BET surface area of Nb-CeO_2_, which is prone to highly spread Au nanoparticles on the surface and increases the contact of the catalyst with reactant molecular. As benzene combustion is an exothermic reaction, the extended reaction sites are easy to avoid heat concentration, which could help to prevent Au nanoparticles aggregation with each other. Furthermore, there are stronger Au–support interaction over Au/Nb-CeO_2_ than Au/CeO_2_, which is beneficial to stabilize nano-Au particles on catalyst surface. Thus, the slightly improved Au dispersion and redox properties resulted in the small increase on initial activity of Au/Nb-CeO_2_, but the large BET surface area and the strong Au–support interaction greatly promoted the high-temperature performance improvement of Au/Nb-CeO_2_ for benzene combustion reaction.

## 4. Conclusions

Au/Nb-CeO_2_ catalyst was obtained by loading Au to Nb-modified CeO_2_ adopting a facile thermal decomposition method. NbO_x_ species are mainly present in Au/Nb-CeO_2_ catalyst in the forms of highly dispersed states and bulk species. The two forms of NbO_x_ species modify properties of the Au/Nb-CeO_2_ by generating lattice distortion and modulating Au–support interaction. Nb addition make Au/Nb-CeO_2_ has a larger surface area, better redox ability and stronger Au–support interaction than Au/CeO_2_. It is also found that Nb addition slightly enhanced the initial activity of Au/CeO_2_, but greatly improved its high-temperature performance. There are a much decreased *T*_90%_ and enhanced stability on Au/Nb-CeO_2_ catalyst than Au/CeO_2_. The slightly improved Au dispersion and redox properties resulted in the small increase on initial activity of Au/Nb-CeO_2_, but the large BET surface area and the strong Au–support interaction greatly promoted the high-temperature performance improvement and durability of Au/Nb-CeO_2_ for benzene combustion reaction.

## Figures and Tables

**Table 1:** Data of characterization of as-obtained catalysts.

Catalyst	Crystalline Size (nm) ^a^	*S_BET_* (m^2^ g^−1^) ^b^	H_2_ Consumption (mmol g^−1^) ^c^	Au (wt%) ^d^	XPS Data			
					Au*^δ^*^+^/Au	Ce^3+^/Ce^4+^	O_sup_/O_latt_	Nb/Ce
Au/CeO_2_	12	71	5.13	0.92	0.32	0.31	0.70	-
Au/Nb-CeO_2_	7	203	5.79	0.99 (0.07) ^e^	0.41	0.32	1.33	0.37
Au/NbO_x_	-	44	-	-	0.12	-	0.65	-
Nb-CeO_2_	-	-	-	-	-	0.17	2.21	0.67

^a^: CeO_2_ Crystalline size calculated by Scherrer equation from index of (111) facet. ^b^: BET surface area. ^c^: calculated from H_2_-TPR profiles at <200 °C. ^d^: ICP result. ^e^: the value in bracket is Nb/Ce molar ratio.

**Table 2:** Full width at half maximum (FWHM) and frequency of the dominant CeO_2_ line in the Raman spectra of the sample.

Catalyst	Frequency (cm^−1^)	FWHM (cm^−1^)
CeO_2_	457	34
Nb-CeO_2_	454	32
Au/CeO_2_	452	51
Au/Nb-CeO_2_	456	65

**Table 3:** Au dispersion of the obtained samples.

Catalysts	Au/CeO_2_	Au/Nb-CeO_2_	Au/CeO_2_-1st ^a^	Au/Nb-CeO_2_-1st	Au/Nb-CeO_2_-48 h ^b^
Au dispersion (%)	68	72	54	72	71
Au particle size ^c^ (nm)	1.72	1.63	2.15	1.63	1.67

^a^: Au/CeO_2_-1st refers to the Au/CeO_2_ after one run. ^b^: Au/Nb-CeO_2_-48 h refers to the Au/Nb-CeO_2_ after 48 h test at 230 °C. ^c^: calculated from CO pulse adsorption.

## Data Availability

The data presented in this study are available on request from the corresponding author.
